# The effectiveness of couple-based interventions on the marital outcomes of women with genital and breast cancer and their partners: a systematic review and meta-analysis

**DOI:** 10.1186/s12885-024-12088-x

**Published:** 2024-03-27

**Authors:** Hamideh Zahedi, Zohreh Alizadeh-Dibazari, Mojgan Mirghafourvand, Mohammad Hasan Sahebihagh, Mina Hosseinzadeh

**Affiliations:** 1 Department of Community Health Nursing, Nursing & Midwifery Faculty, Student Research Committee, Tabriz University of Medical sciences, Tabriz, Iran; 2 Midwifery Department, Faculty of Nursing and Midwifery, Student Research Committee, Tabriz University of Medical Sciences, Tabriz, Iran; 3 Social determinants of Health Research Center, Faculty of Nursing and Midwifery, Tabriz University of Medical Sciences, Tabriz, Iran; 4 Department of Community Health Nursing, Nursing & Midwifery Faculty, Tabriz University of Medical sciences, Tabriz, Iran

**Keywords:** Couple-based interventions, Breast cancer, Genital cancer, Marital adjustment, Marital satisfaction, Marital intimacy, Partner

## Abstract

**Background:**

Breast cancer and genital cancer are known as cancers that affect people’s relationships with their partners. Women with such cancers are emotionally vulnerable and need more support from their partners. The present systematic review and meta-analysis evaluated the effectiveness of couple-based interventions on the marital outcomes of patients with these cancers and their intimate partners.

**Methods:**

To perform this systematic review, Google Scholar and databases such as PubMed, Web of Science, Cochrane, Scopus, SID (Scientific Information Database), and Magiran were searched systematically. The reviewed studies included randomized controlled trials and quasiexperimental studies in which the intervention group, couple-based interventions, and the control group received routine care, general education or no intervention for cancer treatment. In this study, the included participants were patients with breast cancer or genital cancer and their intimate partners. The primary outcomes considered in this study included patients’ marital adjustment, patients’ marital satisfaction, patients’ marital intimacy, and patients’ marital relationships. The secondary outcomes were partners’ marital adjustment, partners’ marital satisfaction, partners’ marital intimacy, and partners’ marital relationships. A meta-analysis was performed with Review Manager v. 5.3 software (The Nordic Cochrane Centre, Cochrane Collaboration, 2014; Copenhagen, Denmark). The intervention impacts on continuous outcomes were measured using standardized mean differences (SMDs) with 95% confidence interval because of the use of various scales to evaluate the outcomes. The quality of evidence presented in the included studies was evaluated using the Grading of Recommendations Assessment, Development, and Evaluation (GRADE) approach. In the subgroup analysis, the studied outcomes were divided into two parts (theory-based and non-theory-based) in terms of the theoretical context of couple-based interventions.

**Results:**

From a total of 138 retrieved studies, 14 trials were eligible for inclusion in the study. The results of the meta-analysis showed that the patient’s marital satisfaction increased significantly with couple-based interventions (SMD 0.46, 95% confidence interval 0.07 to 0.85; 7 trials, 341 patients, very low certainty) compared to the control group, but the evidence was uncertain. However, there were no significant differences between the groups in the partner’s marital satisfaction, the patient’s and partner’s marital adjustment, and the patient’s and partner’s marital intimacy. Additionally, the results of the subgroup analysis showed that the couple-based interventions significantly increased the patient’s marital adjustment (SMD 1.96, 95% CI 0.87 to 3.06; 4 trials, 355 patients, very low certainty), the partner’s marital adjustment (SMD 0.53, 95% CI 0.20 to 0.86; 4 trials, 347 partners, very low certainty), the patient’s marital satisfaction (SMD 0.89, 95% CI 0.35 to 1.43; 2 trials, 123 patients, very low certainty), and the partner’s marital satisfaction (SMD 0.57, 95% CI 0.20 to 0.94; 2 trials, 123 partners, very low certainty) compared to the control group in theory-based studies. In. However, in non-theory-based studies, the results of the meta-analysis revealed no significant differences between the intervention and control groups.

**Conclusions:**

The results of this study demonstrated the impact of couple-based interventions on the marital outcomes of patients with breast and genital cancers. Because of the very low confidence in the evidence, high-quality randomized trials with a sufficient sample size should be conducted considering the proper theoretical context.

**Supplementary Information:**

The online version contains supplementary material available at 10.1186/s12885-024-12088-x.

## Introduction

Breast, uterine, cervical, and ovarian cancers are the most common cancers among women. Breast cancer is highly prevalent in developing and developed countries and accounts for nearly one-third of newly diagnosed cancers in women [1]. With nearly 2.26 million new cases in 2020, breast cancer was identified as the most common women’s cancer worldwide, which includes 12.5% of all cancers in women [2]. In addition to breast cancer, genital cancer is extensively prevalent among women [3]. In 2020, the incidence rate of genital cancers around the world was reported by more than 1.3 million women, 7.29% of whom comprise new cancer cases worldwide [4].

Despite the increasing incidence of cancer, the enhancement of diagnostic and treatment methods has increased the cancer survival rate and the number of affected women, which has affected various people through the long-term diagnostic and treatment of cancer [5]; these findings further clarify the need to focus on patients’ quality of life. Breast and genital cancers lead to broad changes in the personal and marital lives of infected women [6]. The results of a recent study indicated that patients with women-specific cancers experience multiple unfavorable situations, including lowering intimacy with their partners and trying to maintain their sexual exclusivity. Women with breast cancer suffer from problems such as decreased self-esteem, a decreased sense of femininity, weakness in sexual relations, and poor body image because of mastectomy, which disturbs their marital life [7]. This cancer not only creates a severe mental burden for patients but also for their life partners [8]. According to the results of a systematic review, the husbands and male partners of women who suffer from breast cancer experience profound and considerable changes in terms of family life and feelings [9]. Studies indicate that marital problems caused by cancer treatment are common and distressing consequences for individuals with genital and breast cancer. This can lead to changes in their intimate relationships with their partners [10, 11]. This not only affects cancer patients but also their partners in terms of the quality of marital life [12].

Many women are hesitate of talking about their sexual problems, and on the one hand, nurses and doctors disregard this issue; thus, these women deal with this problem alone [11]. Indeed, women with cancer and their partners need considerable intervention to resolve a variety of sexual and marital problems induced by cancer treatment [13]. Considering the psychosocial adaptability and ability of partners to communicate effectively and cope together, there is wide interest in couple-based interventions in cancer care [14]. A couple-based intervention systematically involves the intimate partner and focuses on the couple as a unit. This type of intervention can be beneficial for both patients and their partners who are dealing with cancer and related sexual problems [15].

Research shows that intimate partners can considerably protect and support women with cancer during the treatment and recovery process [16, 17]. Adopting a couple-centered process may not only decrease negative cancer outcomes for both simultaneously but also support their mental growth and mutual flexibility [18].

Couple-based interventions (including both patients and their intimate partners) [19] can be more advantageous for couples [15] than can those with patients only and couple-based coaching interventions (intimate partners help the patients as assistants or coaches) [20]. Such interventions have long-term effects on maintaining behavioral changes and reducing the concerns of intimate partners during daily care activities to support patients [21, 22]. To date, multiple studies have been conducted on couple-based interventions, but some research has shown contradictory results. For example, Zhang et al. [23] reported a considerable effect of couple-based interventions on marital satisfaction, while Price-Blackshear et al. [24] claimed the opposite result and was even harmful. Additionally, Comez et al. [25] and Li et al. [6] showed the positive effect of couple-based interventions on marital adjustment, whereas Fergus et al. [19] observed no effect of the intervention on marital adjustment. Therefore, systematic reviews seem to be required to analyze the effects of such interventions.

While several systematic reviews have been conducted on couple-based interventions among cancer patients [15, 26, 27], the participants in these review studies were not those with women-specific cancer patients, and the intimate partners of the patients were not included in the examination. Additionally, different outcomes have been investigated. Therefore, to address these gaps and since breast and genital cancers have a similar nature and affect women’s femininity and often have unique psychological, emotional, and social implications for women, including impacts on body image and sexuality, this systematic review and meta-analysis studies the effects of couple-based interventions on marital outcomes, including marital adjustment, marital satisfaction, and marital intimacy, on couples with breast and genital cancer (women) and intimate partners.

## Methodology

This systematic review is based on the Cochrane Handbook for Systematic Reviews, and the results are reported according to PRISMA; it is registered on PROSPERO (Registration number: CRD42023453336).

### Search strategy

Systematic searches of databases, including PubMed, Scopus, Web of Science, Cochrane Library، SID (Scientific Information Database), and Magiran, were performed beginning on 30th April 2023 with related keywords to obtain published studies in English and Persian; the search was completed on 5th June 2023 without any date limits. The complete search strategy for each database is presented in Appendix 1. Additionally, the references used in these studies were manually searched to identify additional associated studies not registered by the electronic search. This search was performed in two steps, once at the beginning and exactly before the end (final search) of the study. There were no differences between the studies included in both periods.

### Inclusion and exclusion criteria

All randomized controlled and quasi-experimental trials in English and Persian that investigated the effectiveness of couple-based interventions in patients with breast and genital cancers and their intimate partners to improve marital outcomes were included in this study. The exclusion criteria were abstracts from conferences, study protocols, and studies without related data.

### Participants

Women with breast and genital cancers and their intimate partners were included in this study.

### The type of interventions

The interventions included any type of couple-based interventions with the involvement of patients with breast and genital cancers and their intimate partners. The control group received no intervention or received routine care or general education.

### Study outcomes

The primary outcomes of this study included the patient’s marital satisfaction, the patient’s marital adjustment, the patient’s marital intimacy, and the patient’s marital relationship. Secondary outcomes included the partner’s marital satisfaction, the partner’s marital adjustment, the partner’s marital intimacy, and the partner’s marital relationship.

### Collection and analysis of the data

#### Study selection

**Fig. 1:**
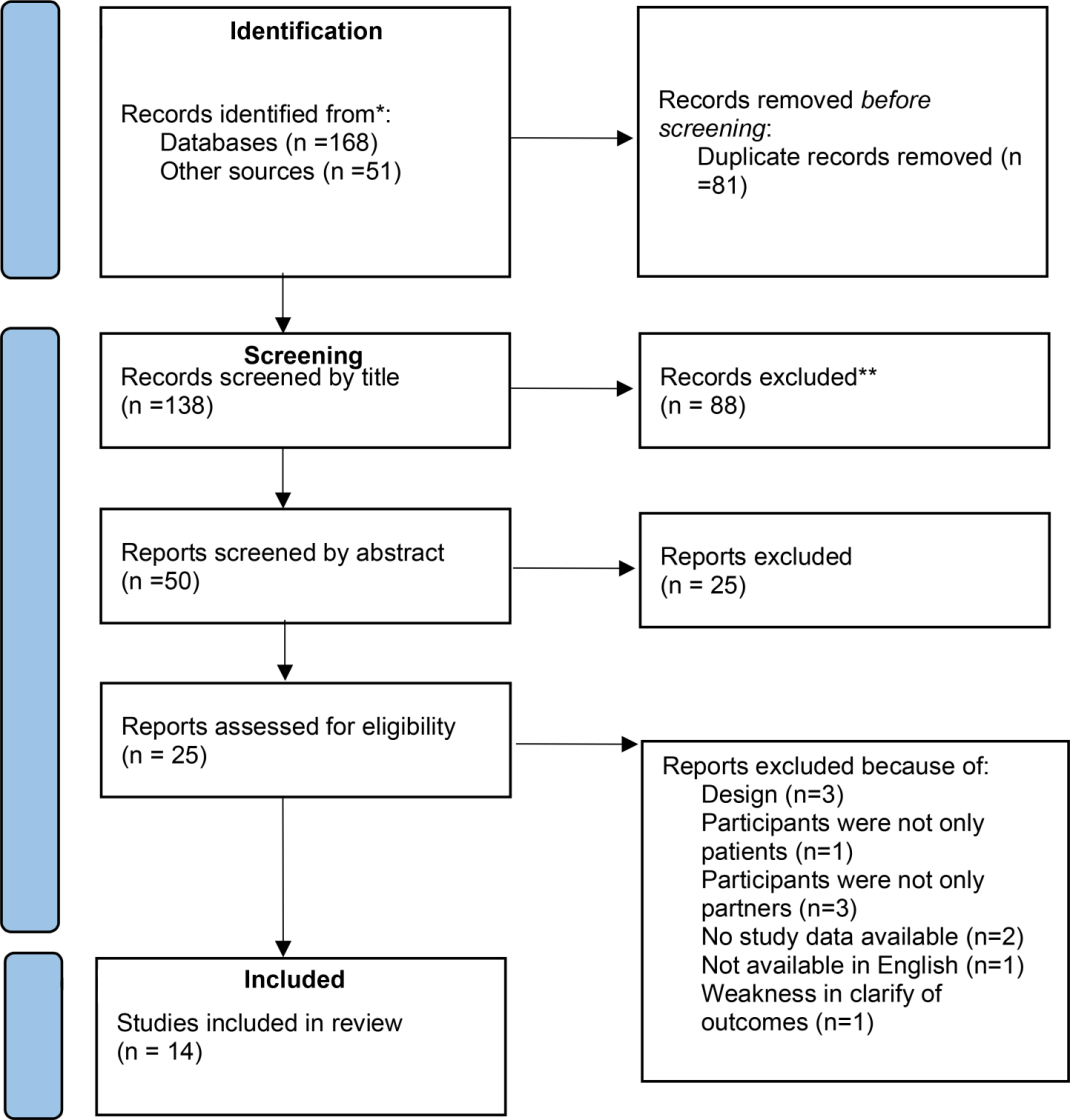
Flow diagram of the systematic review process

EndNote software was used to manage the studies (Clarivate, Thomson Reuters, Philadelphia, Pennsylvania). After removing duplicate cases, two authors, H.Z. and Z.A-D., separately investigated the titles and abstracts of the extracted articles in terms of the inclusion and exclusion criteria, followed by evaluating the full texts of the papers. Any disagreement about the eligibility of the studies was resolved through discussion; otherwise, it was consulted by a third author (M.H.). Figure 1 shows the study flow, the number of identified/excluded studies, and the number of included studies.

### Data extraction and management

To extract data, two authors (H. Z & Z. A-D) extracted the study characteristics independently using a data-extraction form based on the Cochrane Handbook [28]. Any disagreements were resolved through discussion. The extracted data included the first author’s name, country, year of publication, study design, study groups, type of intervention, type of blinding, follow-up period, number of participants in each group, participants’ health status, primary outcomes, secondary outcomes, results, and theoretical contexts.

### Risk of bias assessment in the included studies

Two authors (H.Z & Z. A-D) independently investigated the risk of bias in all included studies using the criteria listed in the Cochrane Handbook. To evaluate the risk of bias in this study, the included randomized controlled trials were investigated by the ROB-1 approach [29] in terms of random sequence generation, allocation concealment, blinding of participants and personnel, blinding of outcome assessors, selective reporting, and incomplete outcome data. In addition, the included semi-experimental trials were reviewed using the ROBINS-1 approach [30]. Then, the judgments were adapted to each other, and any disagreement was resolved by consulting the third author (M.H.).

### The quality assessment of a control set using the GRADE approach

The quality of evidence in the included studies (related to the research outcomes) was evaluated by the GRADE (Grading of Recommendations Assessment, Development, and Evaluation) approach, which includes five dimensions: risk of bias, imprecision, inconsistency, indirectness, and publication bias [31]. This evaluation was independently performed by two authors (H.Z. & Z.A-D.), and any disagreements between the two authors were resolved through discussion with a third author (M.H.). To explore the presence of clinical heterogeneity, all the trials included in the study were described and compared in terms of the studied population’s characteristics and those of interventions offered to the studied groups. The presence of statistical heterogeneity was assessed using the I^2^ statistic and a confidence interval of 95%. In cases with I^2^ ≥ 25%, the certainty of evidence was reduced due to contradictions [32]. To evaluate the indirectness, the study population, type of intervention, control group, and study outcomes were examined in terms of response to the current systematic review [33]. To evaluate the imprecision, the trials were assessed in terms of sufficient participants to calculate the estimation effect and confidence interval around this effect [34]. To compute the quality of evidence for each studied outcome, the quality of evidence was reduced to one and two degrees if there were severe and very severe concerns, respectively.

### Synthesis of results

#### Measures of treatment effect

Data on marital satisfaction, marital adjustment, and marital intimacy were extracted from the patients and intimate partners for the control and intervention groups. To calculate the impacts of the interventions on the continuously studied outcomes of the trials, the mean difference and standard deviation before and after the intervention were first obtained for the intervention and control groups. In addition, a standardized mean difference (SMD) (with a 95% confidence interval) was used to report outcomes using different scales to examine continuous outcomes [35].

### Data analysis

The data were analyzed to compare the study outcomes between the intervention and control groups in cases with at least two trials with Review Manager 5.3 software. In the case of high heterogeneity between the studies (I^2^ ≥ 25%), the random effect method was used rather than the fixed effect method to calculate the size of the intervention impact on the outcome of interest. Regarding the studies the control group did not receive routine care or no intervention, we excluded those studies and did a meta-analysis again. In the subgroup analysis, the studies were divided into two parts (theory-based and non-theory-based) in terms of the theoretical context. The theories used in the included studies are the Roy adaptation model, the systemic transactional model of stress and coping, the PLISSIT model, attachment behavior and attachment style, theories of behavioral couples, and the preliminary live with love conceptual framework.

## Results

### Description of the studies

The results of the search strategy for the studies are summarized in the PRISMA diagram (Fig. 1). From a total of 138 retrieved studies in the searching process, 113 studies were screened and excluded because they did not meet the inclusion criteria for this study. Finally, 14 trials out of 25 reviewed studies were included in the final analysis based on the research target and inclusion criteria (Table 1), with 11 excluded studies (Table 2).

**Table 1:** Characteristics of excluded trials

Author (s) Location/(year)	Study design	Study groups	Type of intervention/s	Type of blinding	Follow-up period	Number of participants in each group	Health status of participants	Main outcome/s	Secondary outcome/s	Results	Theoretical frameworks/theories
Baucom et al. USA/(2009) [36]	Pilot RCT	Group 1: Intervention group Group 2: Control group	Intervention group: Couple-based relationship enhancement (RE) Face-to-face Frequency: 1/2weeks Duration: 12 weeks six sessions of 75 min Delivery with a psychologist Control group: Treatment-as-usual (TAU)	Double-blind	Post-treatment, and 12 months later.	Intervention group: 8 Control group: 6	Women with stage I-II breast cancer, no history of other breast cancer, and no history of cancer within the last 5 years	**Patients**: Relationship functioning (relationship satisfaction and sexual functioning), psychological functioning (brief symptom, posttraumatic growth, functional assessment of cancer therapy, self-image), cancer-related medical symptoms (brief fatigue, brief pain, Rotterdam symptom) **partners**: Relationship functioning (relationship satisfaction and sexual functioning), psychological functioning (brief symptom, posttraumatic growth)		At both posttest and 1-year follow-up, couples who participated in relationship education (RE) showed greater improvements in individual psychological and relationship functioning, as well as relationship satisfaction, compared to those who did not receive RE (TAU). Additionally, women who participated in RE reported experiencing fewer medical symptoms than women in the TAU group.	Non-Clear
Budin et al. New York /(2008) [37]	RCT^a^	Group 1: Intervention group Group 2: Intervention group Group 3: Intervention group Group 4: Control group	Intervention group: 1) Standardized psychoeducation (SE): Four phase-specific psychoeducation videos (coping, recovering from surgery, understanding adjuvant therapy, and recovery) 2) Telephone counseling (TC): Enhance the patient’s and partner’s sense of control and mastery 3) Standardized psychoeducation plus telephone counseling (SE + TC): Four sessions delivery with trained nurses Control group: Standard care (disease management: DM)	Open-label design	Post-surgery, Adjuvant therapy, and Ongoing recovery phases ( 2 weeks after completion of chemotherapy or radiation or 6 months after surgery if no adjuvant therapy was received)	Intervention group for the patient: SE: 45 TC: 42 (SE + TC): 40 Control group for the patient: DM: 50 Intervention group for partners: SE: 34 TC: 30 (SE + TC): 29 Control group for partners: DM: 33	The patients who were diagnosed with a confirmed or strongly suspected breast cancer lesion, and their partners.	Emotional adjustment: Psychological well-being Physical adjustment: Overall health status, physical symptoms Social adjustment: Vocational, domestic, and social environments		The findings of the study showed that the intervention had no statistically significant for patients in groups about psychological well-being (*p* = 0.62), overall health (*p* = 0.82), vocational environment (*p* = 0.052), or social environment (*p* = 0.92). The intervention had a significant difference in the physical symptoms (*p* = 0.024) and vocational environment (*p* = 0.046) of partners.	The theoretical framework was based on the stress and coping model of Lazarus and Folkman, and the crisis intervention model.
Christensen. USA/ (1983) [42]	RCT^a^	Group 1: Treatment group Group 2: Control group	Intervention group: Counseling for couples after a mastectomy Face to face 4 sessions Duration: 6 weeks Frequency: 1/week Delivery with trained counselors Control group: Not Clear	Open-label design	One week post-intervention	Intervention group: 10 Control group: 10	Patients with non-metastatic breast cancer who had undergone surgery at least 2 months before the experiment but no more than 3 months prior and also their partners.	**Patients**: Psychological screening (emotional discomfort), marital adjustment, sexual satisfaction, depression, self-esteem, internal-external locus of control **Partners**: Psychological screening (emotional discomfort), marital adjustment, sexual satisfaction, depression, self-esteem, internal-external locus of control		The treatment increased sexual satisfaction and decreased emotional discomfort for dyads (*p* < 0.05), and decreased depression in patients (*p* < 0.05). The Intervention did not significantly affect marital adjustment between the treatment group’s mean score of 106.15 ± 20.68 and the control group’s mean score of 99.6 ± 17.88.	Non-Clear
Comez and Karayurt. Turkey/(2020) [25]	Quasi-experimental ( pretest-posttest with control group)	Group 1: Intervention group Group 2: Control group	Intervention group: Web-based training (breast cancer and treatment methods, the prevention and management of the symptoms that are related to the treatment, arm-shoulder exercises, sexuality, pregnancy write questions and receive answers) Website Frequency: Available during intervention Duration: 3 months Delivery with research nurses Control group: Routine nursing care	Open-label design	Three months post-intervention	Intervention group: 41 Control group: 42	Primary stage I or stage II breast cancer diagnosis, having mastectomy or breast-conserving surgery in the last 10 days	**Patients**: -Functional assessment of cancer therapy-breast (FACT-B): Life quality -Dyadic adjustment (DAS) **Partners**: -Dyadic adjustment (DAS)		The finding of the study showed that there were significant positive differences in dyadic adjustment among women and their partners in the intervention and control group in the third month after the web-based training (*p* < 0.001). There were significant differences in the life quality of women in the Intervention Group compared with the control group after the web-based training (*p* < 0.05).	The conceptual framework of the present study consisted of the Roy Adaptation Model (RAM).
Fergus et al. Canada/(2022) [19]	Multicenter RCT	Group 1: Treatment group Group 2: Control group	Treatment group: Couple links online intervention with website 6 sessions Duration: 8 weeks Frequency: 1/week Delivery with trained facilitators (five mental health professionals) Control group: Waitlist control (Did not receive any intervention)	Open-label design	Post-intervention, Follow-up: Three months after intervention	Treatment group: 31 Control group: 36	Non-metastatic, invasive breast cancer or ductal carcinoma in situ within the last 36- month	**Patients**: Dyadic coping, dyadic adjustment, marital satisfaction, breast cancer, and relationship **Partners**: Dyadic coping, dyadic adjustment, marital satisfaction, breast cancer, and relationship	**Patients**: Depression and anxiety **Partners**: Depression and anxiety	The treatment group showed a significant improvement in positive dyadic coping (*p* = 0.032), breast cancer, and relationship (*p* = 0.04), but positive effects were not sustained at the 3-month follow-up The treatment group showed a significant decrease in their anxiety levels over time (*p* = 0.03). The study did not observe any impact on the overall relationship adjustment.	Non-Clear
Hedayati et al. Iran/(2020) [41]	pretest-posttest control group design	Group 1: Intervention group Group 2: Control group	Intervention group: Emotionally focused couple therapy (EFT) 6 sessions (120 min per session) Face to face Duration: 6 weeks Frequency: 1/week Control group: Did not receive any intervention	Open-label design	Post Intervention	Intervention group: 11 Control group: 11	Breast Cancer with stage II, post-mastectomy	**Couples**: Marital intimacy		The statistical analysis showed a significant difference in the components of marital intimacy from the pre-test to the post-test (*p* < 0.01). The intervention group exhibited higher mean scores for every component of marital intimacy following their participation in the educational program, as compared to the control group.	Emotionally focused couples therapy taken from the suggestions of Susan Johnson known as HMT (Hold Me Tight)
Jonsdottir (2021) Iceland [17]	quasi-experimental(one-group pre and post setup)	Group 1: Intervention group Group 2: Control group	Intervention group: Couple strengths-oriented therapeutic conservations (CO-SOTC) + web-based evidence-based educational information Three sessions of 45 min Face to face Frequency: 1–2 weeks apart and the final session was conducted 3 months after the first session Duration: 3 months Delivery with nurses Control group: Wait-list (Did not receive any intervention)	Open-label design	Two weeks post-intervention and assessment over three months	Intervention group = 34 Control group: 26	60 couples diagnosed with cancer (regardless of type and stage), currently in active cancer treatment	-Ice-beliefs: Illness beliefs about sexuality and intimacy -Relationship quality -Dyadic difference	------	The CO-SOTC intervention had significant differences in sexuality, intimacy (*p* < 0.001), and overall quality of the relationship (*P* = 0.005) of both women and their partners over time.	The theoretical model of the study is a family strength-oriented therapeutic conversation (FAM-SOTC).
Kalaitz et al. Greece/(2007) [43]	RCT^a^	Group 1: Intervention group Group 2: Control group	Intervention group: a combination of brief couples and sex therapy (CBPI) Face to face 6 sessions Frequency:1/2 weeks Duration: 3 months Delivery with trained therapists Control group: Not clear	Open-label design	Post Intervention	Intervention group: 20 Control group: 20	Underwent simple mastectomy for in situ, primary breast cancer; negative for estrogens/ progesterone receptors; no patient received tamoxifen, chemotherapy, or radiation therapy	**Patients**: Anxiety, depression, sexuality and body image (satisfaction with relationship and satisfaction with body image)		The patients who received the combined brief psychosexual intervention (CBPI) exhibited noteworthy improvements in various aspects of their well-being. Specifically, they showed significant improvement in depression (*p* = 0.013) and state anxiety (*p* = 0.006), as well as in body image, satisfaction with their relationship (*p* < 0.001), presumed attractiveness to their partner, frequency of orgasms (*p* = 0.042), and communication about their desires.	Non-Clear
Li et al. China/ (2023) [6]	RCT^a^	Group 1: Intervention group Group 2: Control group	Intervention group: WeChat couple-based psychosocial support (articles about psychoeducation, skills training, and counseling) Session: Not Clear Frequency: 1/2days Duration: 8 weeks Control group: Received six WeChat articles on general education content related to diet and exercise	Open-label design	Post-intervention and three months after the intervention.	Intervention group: 49 Control group: 49	women with a newly diagnosed gynecological cancer (ovarian, uterine, cervical, vulvar, and vaginal cancer) 6 weeks after any surgery	**Patients**: Sexual function	**Patients and partners**: Dyadic adjustment and quality of life	It was found that the dyadic adjustment was improved significantly in the intervention program in women (*p* = 0.001) and their partners (*p* = 0.027). The intervention program also demonstrated statistically significant positive effects on the quality of life of women with gynecological cancer (*p* = 0.035).	Systemic Transactional Model of Stress and Coping and effective couple-based intervention approaches.
Nho et. South Korea /(2019) [38]	Quasi-experimental (pretest-posttest design)	Group 1: Intervention group Group 2: Control group	Intervention group: Web-based sexual health enhancement program (WSHEP) (five modules and each module consisted of between one and six interventions) Five sessions Frequency: Not clear Duration: Four week Delivery with nurses Control group: underwent the current standard of care routinely provided in the clinic	Open-label design	After 4 months	Intervention group: 21 couples Control group: 22 couples	-Women with Stage I-II gynecological cancer -Completed cancer treatment -No contraindication for sexual intercourse	**Patients**: Sexual function, sexual distress, and marital intimacy **Partners**: Marital intimacy	------	WSHEP had a significant improvement in all dimensions of sexual function (*p* < 0.001) of women and in the marital intimacy of husbands (*p* = 0.015)	The framework was designed using intervention strategies based on the PLISSIT model.
Nicolaisen et al. Denmark/ (2018) [39]	Multicenter RCT	Group 1: Intervention group Group 2: Control group	Intervention group: Hand-in-hand (HiH) intervention + usual care (enhance dyadic adjustment through dyadic coping within the couples) Face to face 4–8 sessions Frequency: Not clear Duration: up to 5 months after primary surgery Delivery with clinical psychologists Control group: Usual care (verbal and written information on normal psychological reactions about a cancer diagnosis)	Single blind	Post-intervention (five months after surgery), follow-up (10 months after surgery)	Intervention group: 82 Control group: 64	newly diagnosed with primary breast cancer had received no neo-- adjuvant treatment	Patients’ cancer-related distress	Cancer-related distress, symptoms of anxiety and depression, dyadic adjustment	HiH intervention did not significantly affect Cancer-related distress at post-intervention (*p* = 0.08 or follow-up (*p* = 0.71). There was a positive significant difference in the level of dyadic adjustment at follow-up for both patients (*p* = 0.04) and partners (*p* = 0.02).	The theoretical framework attachment theory explains how attachment behavior and attachment style may influence the exchange of support within couples and their adjustment to BC.
Price-Blackshear et al. USA/(2020) [24]	RCT^a^	Group 1: Intervention group Group 2: Control group	Intervention group: CMBI (couples mindfulness-based intervention) 8 sessions of one-hour prerecorded videos Frequency: 1/week Duration: 8 weeks Delivery with trained MBSR teachers Control group: I-MBI (Individual mindfulness-based intervention)	Open-label design	Post Intervention	Intervention group: 36 Control group: 41	One-year post-diagnosis; within 6 years of diagnosis; breast cancer stages 0- III	Perceived stress, depression, and anxiety, The mindful attention and awareness	Dyadic adjustment, relationship satisfaction, interpersonal mindfulness	Levels of perceived stress and anxiety were lower after the intervention in both C-MBI and I-MBI groups (*p* < 0.001). The dyadic adjustment was lower for patients (Baseline Mean = 98.54, Post-intervention Mean 95.50) and relatively no change for partners (Baseline Mean = 100.78, Post-intervention Mean = 101.71) in the C-MBI condition. Relationship satisfaction was lower for patients (Baseline mean = 35.26, Post-intervention Mean = 34.12) and their partners (Baseline Mean = 37.04, Post-intervention Mean = 35.67) in the C-MBI condition.	Non-Clear
Reese et al. USA/(2018) [40]	Pilot RCT	Group 1: Intervention group Group 2: Control group	Intervention group: Couple-based intervention, Intimacy enhancement (IE) (evidence-based sexuality interventions) via Telephone 4 sessions of 60–75 min Frequency: 1/week Duration: 4 weeks Delivery with trained psychosocial providers Control group: Usual care 4 session	Open-label design	Post-intervention	Intervention group: 19 Control group: 9	Had completed active treatment 6 months − 5 years ago for non-recurrent Stage I–III breast cancer (current use of endocrine therapy was acceptable	**Patients and partners**: -Sexual outcomes: Sexual function, sexual satisfaction, sexual distress, and self-efficacy -Relationship outcomes: Dyadic sexual communication, emotional intimacy, dyadic adjustment, -Psychosocial outcomes: cancer-related distress, body image distress, depressive symptoms and anxiety		The intervention had a large effect on sexual satisfaction (Effect size 1.75) in women and a medium effect among their partners (Effect size 0.52). Regarding relationship outcomes, there was no change in emotional intimacy (Effect size = 0.04) among women and a small effect on their partners (Effect size= -0.44). Also in psychosocial outcomes, there was a large effect for a reduction in anxiety symptoms (Effect size= -1.36) among women.	Theories of behavioral Couples.
Zhang et al. China/(2022) [23]	RCT^a^	Group 1: Intervention group Group 2: Control group	Intervention group: Nurse-led couple intervention + routine nursing care (recognizing and facing up to family and marital problems, enhancing communication skills, and learning to solve intimate relationship issues) Four sessions of one hour Frequency: 1/month Duration: 4 months Delivery with trained nurses Control group: Routine nursing care	Single-blind	Two months post-intervention and three months post-intervention	Intervention group: 46 Control group: 49	Confirmed diagnosis of gynecological cancer (ovarian tumor, endometrial carcinoma, and cervical cancer) at I-IV FIGO stage, undergoing surgery and/or periodic radiotherapy and/or chemotherapy	**Patients and partners**: Marital quality: Marital satisfaction, marital communication, and sexual life		The intervention group consisting of patients and their husbands reported significantly improved scores in marital satisfaction (*P* = 0.028). The intervention did not have a significant impact on patient-reported or husband-reported sexual life (*P* = 0.073).	The Preliminary Live with Love Conceptual Framework (P-LLCF).

^a^ Randomized Controlled Trial

**Table 2:** Characteristics of excluded trials. The main reason for exclusion

**Differences in intervention participants**	
Bultz et al. 2000 [54] Lewis et al.2019 [20] Razavi et al.2000 [55]	Participants in the educational intervention were only partners
Shahed et al.2016 [56]	Participants in the educational intervention were only patients
**Differences in methodology**	
Naghiyaee et al. 2014 [57]	Single-case experimental design
Harb et al.2022 [58]	A Mixed-Methods Integrative Study
Manne et al.2004 [59]	Correlational study
**Weakness in clarity of outcomes**	
Scott et al.2004 [60]	The sexual adjustment scale is not clearly stated.
**No study Data**	
Suzuki et al.2020 [61]	Lack of access to the full text of the article
Zimmermann et al.2016 [62]	Lack of access to the full text of the article
**The language of the article**	
Nho et al.2013 [63]	Writing an article in Korean

### Characteristics of included studies

The characteristics of the trials included in the systematic review, including the first author’s name, country, year of publication, study design, study groups, type of intervention, type of blinding, follow-up period, number of participants in each group, participants’ health status, main outcomes, secondary outcomes, results, and theoretical contexts, are summarized in Table 1.

The 14 studies comprised randomized controlled trials (RCTs, *n* = 6), multicenter RCTs (*n* = 2), pilot RCTs (*n* = 2), and quasi-experimental (*n* = 4). These studies concentrated on women with breast and genital cancers and their intimate partners. The sample volume (couples included in the study) was 2192 participants (628 and 468 subjects in the intervention and control groups, respectively). These studies were performed in the USA (*n* = 5) and China (*n* = 2), as well as in Greece, Canada, Denmark, Turkey, Iceland, South Korea, and Iran, each with one study. Additionally, nine out of the 14 included studies contained a theoretical context for the intervention. The studies were published in English from 1983 to 2023, except for one study published in Persian.

### Characteristics of couple-based interventions

In all 14 trials included, couple-based interventions were provided as an intervention along with routine care during cancer treatment, and the control group received routine care, general education or no intervention. In the intervention conditions of these trials, couple-based interventions were provided by trained nurses, clinical psychologists, therapists, advisers, and mental health professionals to women with breast and genital cancers and their intimate partners. The intervention duration ranged from 4 weeks to 4 months, and the intervention was carried out in 3–8 sessions. The number of participants in each educational session ranged between 8 and 82, and the duration of each session ranged from 45 to 120 min. The frequency of sessions was different between once and twice a week or once a month. These interventions were implemented as face-to-face, educational videos, telephone advice through a website, and the sending of educational articles on an Internet platform. The provided educational content included enhancing relationships, adjuvant treatment, postoperative recovery, promoting a sense of control and the patient’s/life partner’s dominance, breast cancer and treating methods, preventing and managing treatment-related symptoms, arm and shoulder exercises, pregnancy, therapeutic conversation based on couples’ strengths, mental education, skill training, consultation, knowing and dealing with family and marital problems, enhancing relationship skills, and learning problem solving related to intimate relationships.

In the trials included in this study, the control group received routine care in seven studies [23, 25, 36–40]. Three studies contained no intervention control group [17, 19, 41]. In one study, a control group received individualized training [24]. In another study, the control group received general education about diet and exercise [6]. Two other studies did not explicitly report the intervention type received by the control group [42, 43].

The participants included in this systematic review were women with breast and genital cancers and their intimate partners. The participants provided informed consent to participate in the trials, and the descriptions of the articles indicated the participants’ consent for randomization. In a study by Hedayati et al. [41], the “marital intimacy” outcome was reported for couples but not for a patient and the intimate partner separately. The first author was asked for the expected consequence of a patient and partner separately, but no response was received. In a study by Kalaitzi, the “marital satisfaction” outcome was only reported for the patient, not for the intimate partner [43].

### Risk of bias in the included studies

**Fig. 2:**
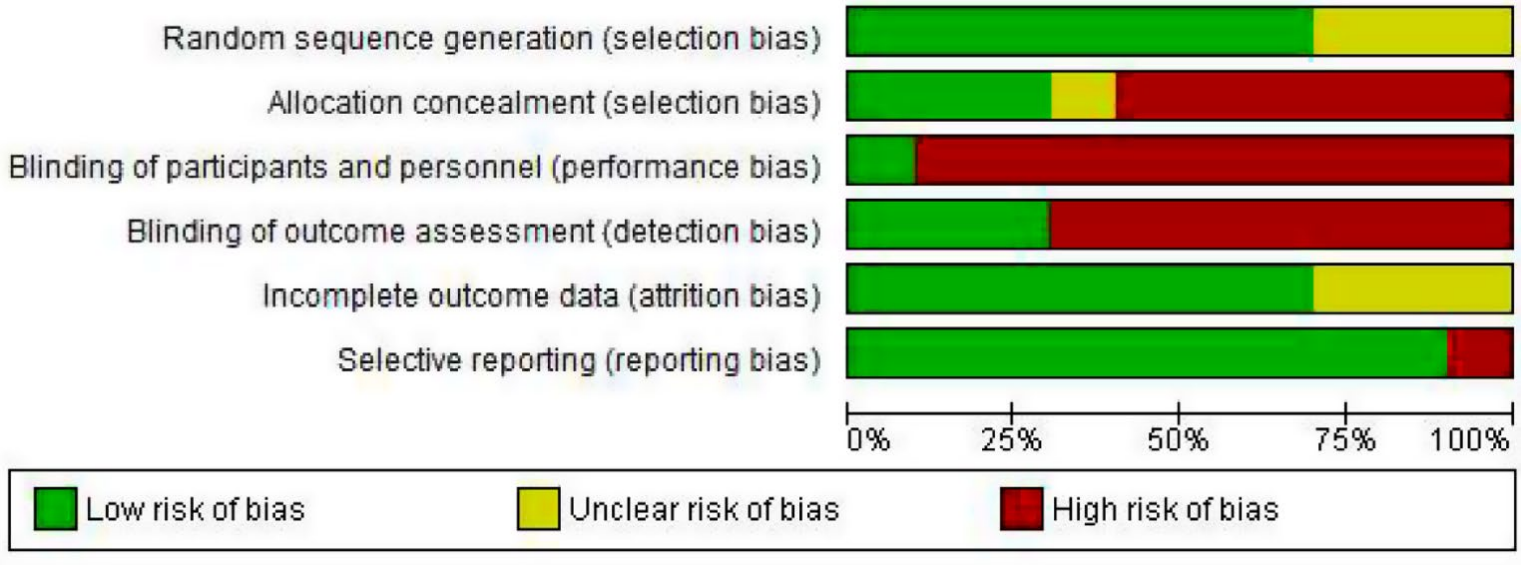
Risk of bias graph. Review authors’ judgments about each risk of bias item presented as percentages across all included studies

**Fig. 3:**
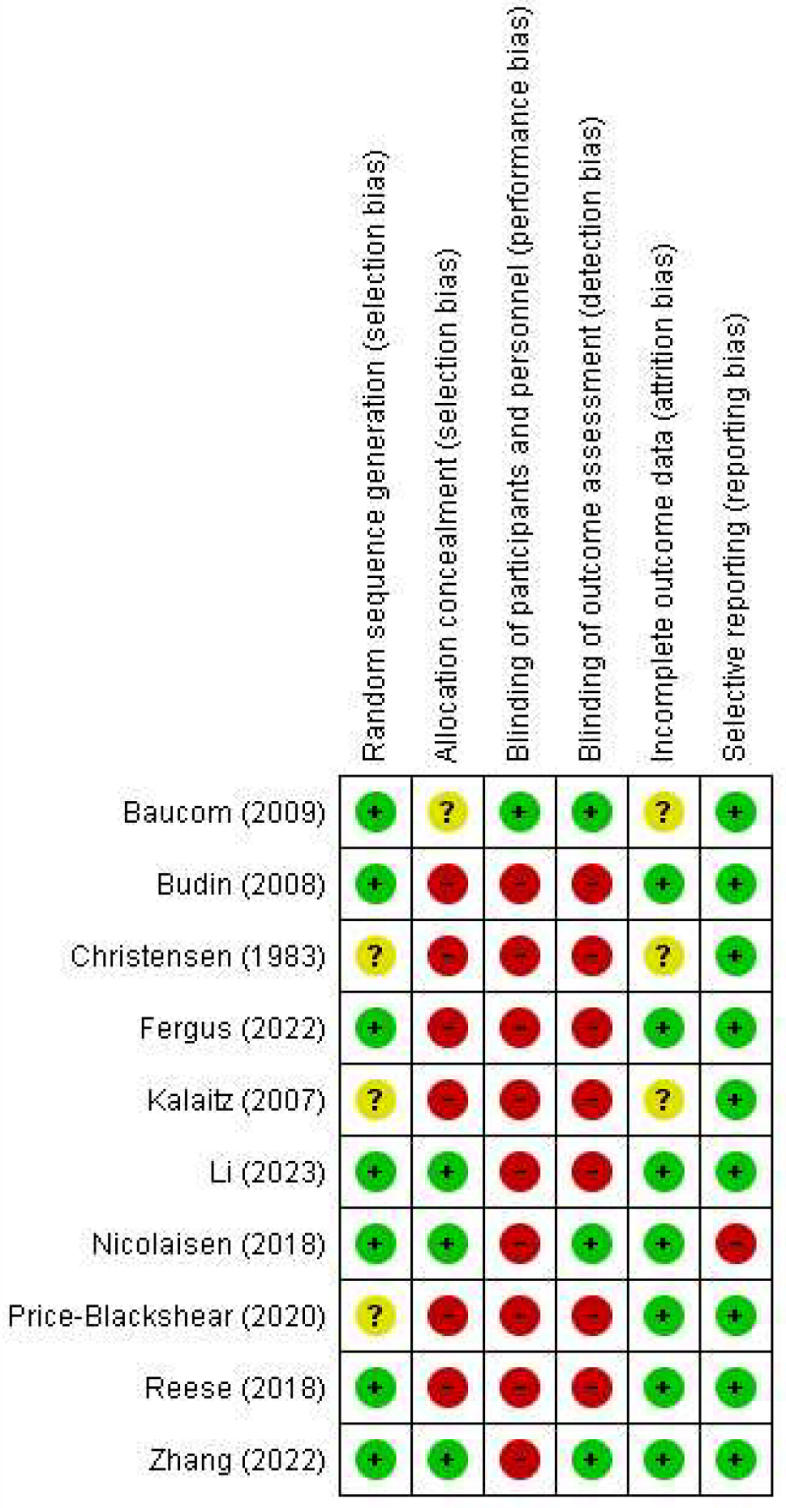
Risk of bias summary: Review authors’ judgments about each risk of bias item for each included study

The evaluation of the quality of the RCTs included in this study is reported in Figs. 2 and 3. All RCTs included in the study were rated as low risk in terms of random sequence generation, except for three cases as an unknown risk [24, 42, 43]. In terms of allocation concealment, however, only three studies were rated as low risk [6, 23, 39], and the remaining studies were rated as high risk or unknown. Based on the nature of the study, i.e., couple-based interventions, blinding the participants and personnel was difficult. Therefore, the participants of the study were only blinded in one study where both the couples and the assessor were blinded to the intervention [37]. The outcome assessors were blinded only in three studies [23, 36, 39], and the remaining were at a high risk. In terms of incomplete outcome data or attrition bias, all studies were rated as low risk, and only three studies were rated as unknown risk [36, 42, 43]. In terms of selective reporting bias, all studies were rated as low risk, and only one study was rated as high risk [39] (see Table 3; Figs. 2 and 3).

**Table 3:** Risk of bias of included studies (RCTs)

Bias	Authors’ judgment	Support for judgment	
**Baucom et al. (2008)**			
Random sequence generation	Low risk	Participants were allocated into interventions and control groups, using a computer-based random number generator.	
Allocation concealment	Unclear risk	Allocation in the groups was done by an employee, but nothing was mentioned about the employee being blind.	
Blinding of participants and personnel	Low risk	Blinding	
Blinding of outcome assessors	Low risk	Blinding	
Incomplete outcome data	Unclear risk	There is not enough information about incomplete data.	
Selective reporting	Low risk	Protocol is not available but it is clear that all pre-specified and expected outcomes of interest are reported.	
**Budin et. (2008)**			
Random sequence generation	Low risk	Participants were allocated into intervention and control groups, using the block randomization method.	
Allocation concealment	High risk	There is not enough information in this regard.	
Blinding of participants and personnel	High risk	Open-label design	
Blinding of outcome assessors	High risk	Open-label design	
Incomplete outcome data	Low risk	21 of 66 patients in intervention group one 24 of 66 patients in intervention group two and 18 of 58 patients in the intervention group three and 9 of 59 patients in the control group were excluded. 32 of 66 partners in intervention group one 36 of 66 partners in intervention group two and 29 of 58 partners in the intervention group three and 26 of 59 partners in the control group were excluded. Reasons for missing data were that interventions were not completed within the specified time frame, patients or partners did not return completed questionnaires, and patients or partners decided to withdraw.	
Selective reporting	Low risk	Protocol is not available but pre-specified outcomes of interest to the review are reported in a pre-specified way.	
**Christensen (1983)**			
Random sequence generation	Unclear risk	It is mentioned in the text that the groups are allocated randomly, but the authors did not provide enough information in this regard.	
Allocation concealment	High risk	There is not enough evidence in this regard.	
Blinding of participants and personnel	High risk	Open-label design	
Blinding of outcome assessors	High risk	Open-label design	
Incomplete outcome data	Unclear risk	There is not enough information about incomplete data.	
Selective reporting	Low risk	Protocol is not available but it is clear that all pre-specified and expected outcomes of interest are reported.	
**Fergus et al. (2022)**			
Random sequence generation	Low risk	Participants were allocated into interventions and control groups, using a randomized block design.	
Allocation concealment	High risk	There is not enough evidence in this regard.	
Blinding of participants and personnel	High risk	Open-label design	
Blinding of outcome assessors	High risk	Open-label design	
Incomplete outcome data	Low risk	Eight of 39 participants in the intervention group were excluded which reasons for missing data are not related to outcomes.	
Selective reporting	Low risk	Protocol is not available but all pre-specified outcomes of interest to the review are reported in the pre-specified way.	
**Kalaitz et al. (2007)**			
Random sequence generation	Unclear risk	It is mentioned in the text that the groups are allocated randomly, but the authors did not provide enough information in this regard.	
Allocation concealment	High risk	There is not enough evidence in this regard.	
Blinding of participants and personnel	High risk	Open-label design	
Blinding of outcome assessors	High risk	Open-label design	
Incomplete outcome data	Unclear risk	There is not enough information about incomplete data.	
Selective reporting	Low risk	Protocol is not available but it is clear that all pre-specified and expected outcomes of interest are reported.	
**Li et al. (2023)**			
Random sequence generation	Low risk	Participants were allocated into interventions and control groups, using a computer random number generator.	
Allocation concealment	Low risk	Allocation concealment was done by sequentially numbered, opaque, sealed envelopes.	
Blinding of participants and personnel	High risk	No blinding	
Blinding of outcome assessors	High risk	No blinding	
Incomplete outcome data	Low risk	14 of 49 participants in the intervention group and 12 of 49 participants in the control group dropped out of the study but reasons for missing data were not related to outcome.	
Selective reporting	Low risk	Protocol is available and all pre-specified outcomes of interest to the review are reported in the pre-specified way.	
**Nicolaisen et al. (2018)**			
Random sequence generation	Low risk	Participants were allocated into interventions and control groups, using the computer-based randomization and block randomization methods.	
Allocation concealment	Low risk	Block size and allocation sequence were performed by independent statisticians.	
Blinding of participants and personnel	High risk	Participants were not blinded	
Blinding of outcome assessors	Low risk	Blinding	
Incomplete outcome data	Low risk	22 of 102 participants in the intervention group and 35 of 96 participants in the control group dropped out of the study but reasons for missing data were not related to outcome.	
Selective reporting	High risk	Protocol is available but all pre-specified outcomes of interest to the review are not reported in the pre-specified way.	
**Price-Blackshear et al. (2020)**			
Random sequence generation	Unclear risk	It is mentioned in the text that the groups are allocated randomly, but the authors did not provide enough information in this regard.	
Allocation concealment	High risk	There was no evidence for allocation concealment.	
Blinding of participants and personnel	High risk	Open-label design	
Blinding of outcome assessors	High risk	Open-label design	
Incomplete outcome data	Low risk	25 of 61 participants in the intervention group and 16 of 57 participants in the control group dropped out of the study. Missing data were not balanced across groups, but the reasons were similar (watched less than 4 videos, too sick, had baby, partner stopped participating, too much time).	
Selective reporting	Low risk	Protocol is not available but it is clear that all pre-specified and expected outcomes of interest are reported.	
**Reese et al. (2018)**			
Random sequence generation	Low risk	Participants were allocated into interventions and control groups, using the stratified and block method	
Allocation concealment	High risk	Study project manager assigned participants to interventions but nothing was mentioned about being blind.	
Blinding of participants and personnel	High risk	Open-label design	
Blinding of outcome assessors	High risk	There is not enough evidence in this regard.	
Incomplete outcome data	Low risk	One of 20 participants in the intervention group was excluded which reasons for missing data are not related to outcomes.	
Selective reporting	Low risk	Protocol is not available but all pre-specified outcomes of interest to the review are reported in the pre-specified way.	
**Zhang et al. (2022)**			
Random sequence generation	Low risk	Participants were allocated into interventions and control groups, using a computer random number generator.	
Allocation concealment	Low risk	Allocation concealment was done by opaque sealed envelopes with group allocation codes	
Blinding of participants and personnel	High risk	No blinding	
Blinding of outcome assessors	Low risk	Blinding	
Incomplete outcome data	Low risk	Five of 51 participants in the intervention and four of 53 participants in the control group were excluded. The reasons for missing data were the 3-month follow-up period due to withdrawal of consent and loss of follow-up	
Selective reporting	Low risk	Protocol is not available but all pre-specified outcomes of interest to the review are reported in the pre-specified way.	

The overall risk of bias in quasi-experimental trials was considered serious due to at least a serious bias in the study subdomains. In terms of bias due to confounding, two studies were at moderate risk [25, 38], one study was at serious risk [41], and one study was at low risk [17]. In terms of bias in the selection of participants, except for one low-risk study [17], the other included studies were at serious [25, 41] or moderate [38] risk. In terms of bias in the classification of interventions, only one study was rated at moderate risk [41], and the others were rated at low risk. In terms of bias due to deviations from intended interventions, all the studies were considered low risk. Regarding bias due to missing data, two studies were at low risk [17, 25], one study was at moderate risk [38], and one study was at no information [41]. All the studies were at serious risk because of bias in the measurement of outcomes. In terms of bias in the selection of reported results, all studies were at moderate risk. In summary, all the quasi-experimental trials included in this study were at serious risk of bias (Table 4).

**Table 4:** Risk of bias of included studies (Semi‑experimental study)

Author	Comez and Karayurt (2020)	Hedayati et al. (2020)	Jonsdottir et. (2021)	Nho et. (2019)			
Bias due to confounding	Moderate	Serious	Low	Moderate			
Bias in selection of participants	Serious	Serious	Low	Moderate			
Bias in the classification of interventions	Low	Moderate	Low	Low			
Bias due to deviations from intended interventions	Low	Low	Low	Low			
Bias due to missing data	Low	No information	Low	Moderate			
Bias in measurement of outcomes	Serious	Serious	Serious	Serious			
Bias in selection of reported result	Moderate	Moderate	Moderate	Moderate			
Overall	Serious	Serious	Serious	Serious			

Low: Low risk of bias (the study is comparable to a well-performed randomized trial with regard to this domain); Moderate: Moderate risk of bias (the study is sound for a non-randomized study with regard to this domain but cannot be considered comparable to a well-performed randomized trial); Serious: Serious risk of bias (the study has some important problems);

## Outcome measurement

### Primary outcomes

#### Marital adjustment of patients

**Fig. 4:**
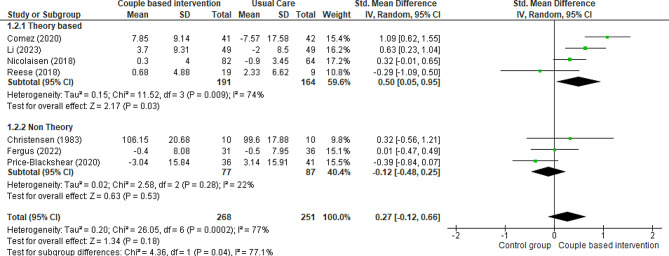
Couple-based intervention group versus control group, Outcome 1: Marital adjustment of patients

Seven RCTs [6, 19, 24, 37, 39, 40, 42] and one quasi-experimental trial [25] compared patients’ marital adjustment in two groups: intervention (receiving couple-based education) and control (receiving routine care or general education or waitlist). Three studies used the Revised Dyadic Adjustment Scale (RDAS) [6, 19, 39], one study utilized the Locke- Wallace Marital Adjustment Test (MAT) [42], one study employed the Dyadic Adjustment Scale [24], one study applied the Dyadic Adjustment Scale (DAS-7) [40], and another used the PAL-C Scale [37]. The results of two studies showed that providing couple-based interventions for couples could positively affect the marital adjustment of patients compared to the control group [6, 39]. On the other hand, two studies indicated the opposite result, that is, a partial decrease in patients’ marital adjustment [24, 40]. In two other studies, couple-based interventions had no effect on patients’ marital adjustment [19, 42]. All these studies were included in the meta-analysis, except for one study by Budin et al., who separately evaluated emotional, physical, and social compatibility in patients with breast cancer and their partners [37]. The results from seven studies conducted on 519 patients indicated that couple-based interventions did not affect marital adjustment compared to routine care, but the evidence is uncertain (Fig. 4) (SMD 0.27, 95% CI -0.12 to 0.66; 7 trials, 519 patients, very low certainty). The result of meta-analysis with excluding studies that the control group received general education showed that there was no change in the significance (SMD 0.33, 95% CI -0.34 to 0.51; 5 trials, 344 patients, very low certainty). The subgroup analysis results showed that theory-based couple-based interventions significantly increased patients; marital adjustment compared to the control group (SMD 0.5, 95% CI 0.05 to 0.95; 4 trials, 355 patients, very low certainty). In contrast, non-theory-based interventions did not significantly influence the patients’ marital adjustment compared to the control group (SMD − 0.12, 95% CI -0.48 to 0.25; 3 trials, 164 patients, very low certainty).

#### Marital satisfaction of patients

**Fig. 5:**
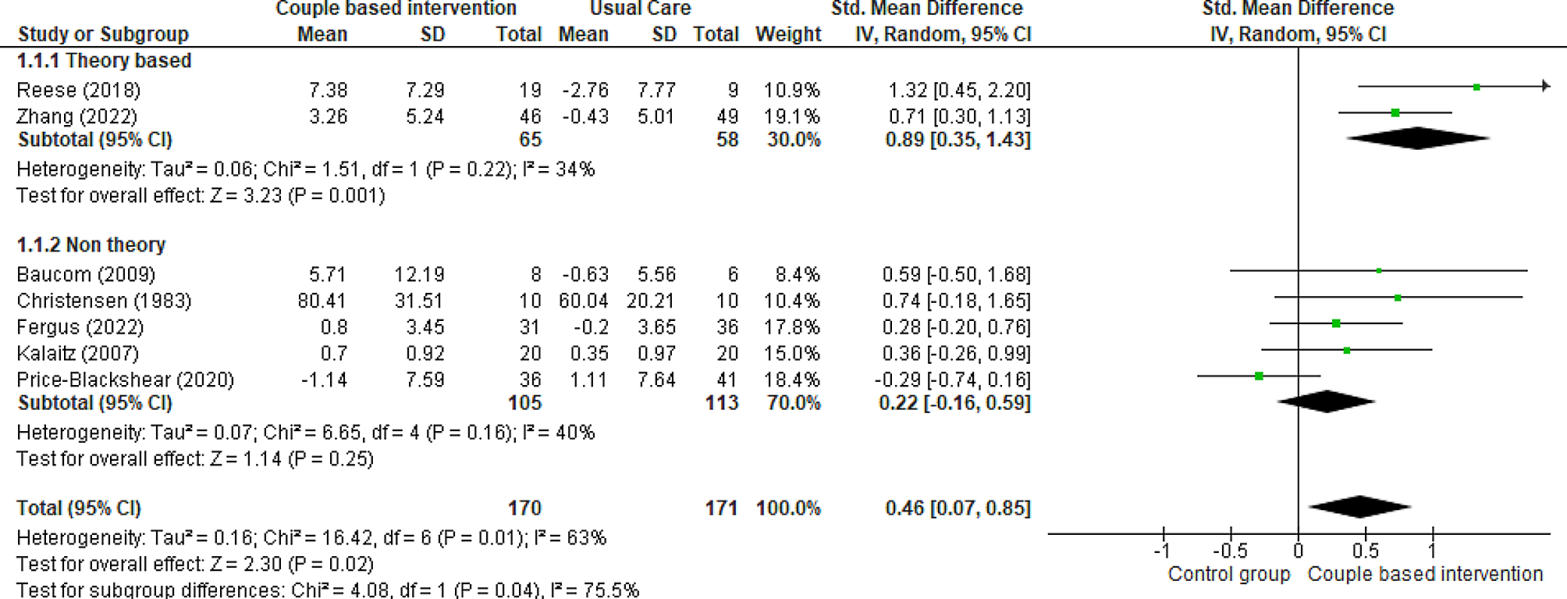
Couple-based intervention group versus control group, Outcome 2: Marital satisfaction of patients

Seven RCTs [19, 23, 24, 36, 40, 42, 43] compared patients’ marital satisfaction in both the intervention (receiving couple-based interventions) and control (receiving routine care or general education or waitlist) groups. To evaluate marital satisfaction, Fergus et al. used the Kansas Marital Satisfaction Survey [19], Zhang et al. utilized the Olson Marital Quality Questionnaire [23], two studies employed the Quality of Marriage Index (QMI) [24, 36], Reese et al. applied the PROMIS SexFS [40], Christensen et al. used the Sexual Satisfaction Scale (SSS) [42], and Kalaitzi et al. utilized the Sexuality and Body Image Scale [43]. Studies showed that couple-based interventions could improve the marital satisfaction of patients [43] compared to the control group [23, 36, 40, 42]. However, the results of one study revealed no change in the patient’s marital satisfaction [19], and another study indicated the opposite effect [24]. All these studies were included in the meta-analysis. The results of seven studies conducted on 341 couples indicated that providing couple-based interventions with routine care might increase patients’ marital satisfaction compared to the control group, but the evidence is uncertain (Fig. 5) (SMD 0.46, 95% CI 0.07 to 0.85; 7 trials, 341 patients, very low certainty). The result of the meta-analysis with excluding studies that the control group received general education showed that there was no change in the significance (SMD 0.59, 95% CI 0.33 to 0.85; 6 trials, 264 patients, very low certainty).

Additionally, the subgroup analysis results showed that theory-based couple-based interventions significantly increased patients’ marital satisfaction compared to the control group (SMD 0.89, 95% CI 0.35 to 1.43; 2 trials, 123 patients, very low certainty). In contrast, non-theory-based couple-based interventions did not significantly influence patients’ marital satisfaction compared to the control group (SMD 0.22, 95% CI -0.16 to 0.59; 5 trials, 218 patients, very low certainty).

#### Marital intimacy of patients

**Fig. 6:**
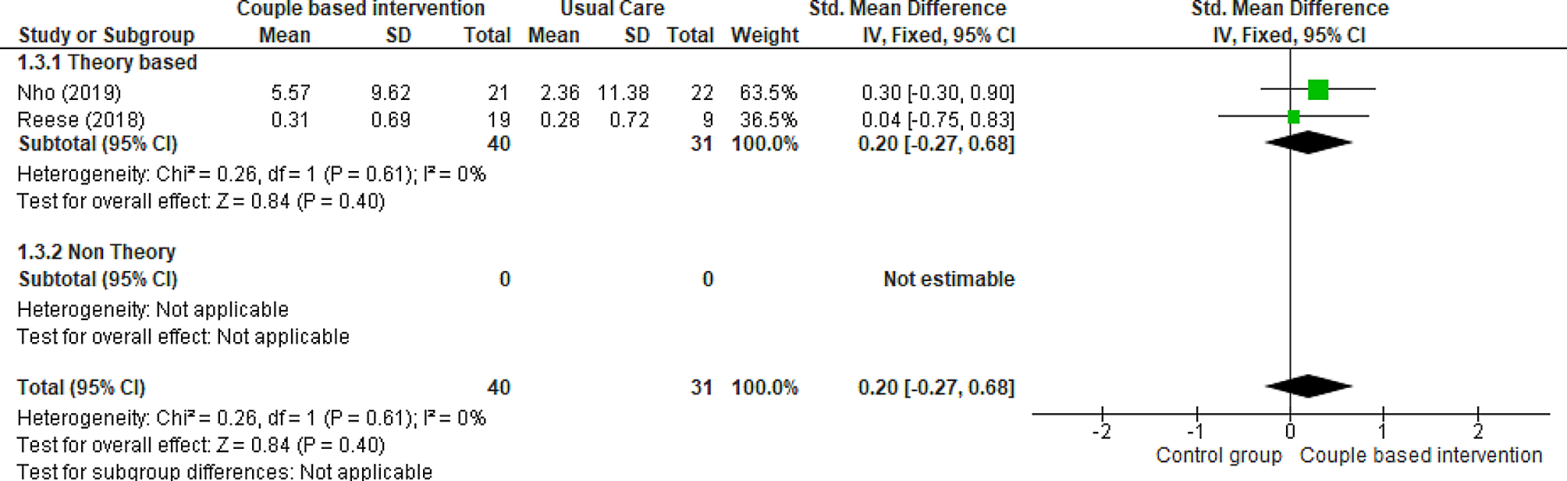
Couple-based intervention group versus control group, Outcome 3: Marital Intimacy of patients

One RCT [40] and three quasi-experimental trials [17, 38, 41] compared the patients’ marital intimacy in the intervention (receiving couple-based intervention) and control (receiving routine care) groups. To evaluate marital intimacy, Reese et al. used the PAIR questionnaire [40], Jonsdottir et al. utilized the Ice-Beliefs questionnaire [17], Nho et al. employed the Marital Intimacy questionnaire [38], and Hedayati et al. applied the Marital Intimacy Questionnaire Bagarozzi [41]. The results of two studies showed that couple-based interventions significantly increased the marital intimacy of patients [17, 41]. On the other hand, Reese et al. reported no significant changes in women’s marital intimacy despite providing couple-based interventions [40]. Although Hedayati et al. reported marital intimacy based on couples but not separately by patients and intimate partners. Additionally, Jonsdottir et al. did not report the results of the control and intervention groups separately. This means that the data of both the control and intervention groups were reported as integrated. Thus, this study was not included in the meta-analysis. A meta-analysis of data from two trials of 71 patients indicated that couple-based interventions did not affect the marital intimacy of patients compared to that of routine care, but the evidence is uncertain (Fig. 6) (SMD 0.20, 95% CI -0.27 to 0.68; 2 trials, 71 patients, very low certainty).

### Marital relationship of patients

No studies were found regarding the effect of couple-based interventions on marital relationships.

### Secondary outcomes

#### Marital adjustment of partner

**Fig. 7:**
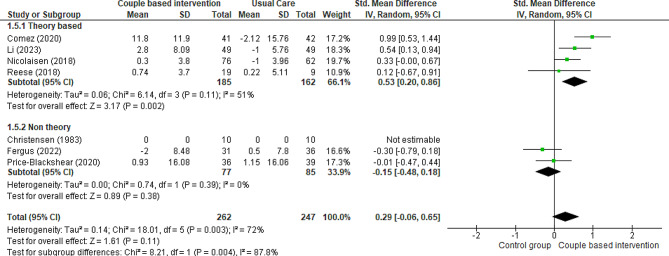
Couple-based intervention group versus control group, Outcome 4: Marital adjustment of partners

Seven RCTs [6, 19, 24, 37, 39, 40, 42] and one quasi-experimental trial [25] compared the marital adjustment of intimate partners in both intervention (receiving couple-based education) and control (receiving routine care or general education or waitlist) groups. Three studies used the Revised Dyadic Adjustment Scale (RDAS) [6, 19, 39], one used the Locke- Wallace Marital Adjustment Test (MAT) [42], one study employed the Dyadic Adjustment Scale [24], one research applied the Dyadic Adjustment Scale (DAS-7) [40], and Budin et al. used the PAL-C [37]. The results of two studies revealed a positive and significant effect of couple-based interventions on the marital adjustment of intimate partners compared with the control group [6, 39]. Additionally, one study reported no change in the marital adjustment of intimate partners [24], and another reported a partial increase [40]. In two other studies, couple-based interventions did not affect the marital adjustment of intimate partners [19, 42]. All the studies were included in the meta-analysis, except for one study evaluating various outcomes [37]. Data obtained from seven studies performed on 509 partners showed that marital adjustment of partners was not influenced by couple-based interventions compared to routine care (Fig. 7) (SMD 0.29, 95% CI -0.06 to 0.65; 7 trials, 509 partners, very low certainty.( The result of the meta-analysis with excluding studies that the control group received general education showed that there was no change in the significance (SMD 0.30, 95% CI -0.24 to 0.84; 5 trials, 336 patients, very low certainty). However, the subgroup analysis showed that theory-based couple-based interventions significantly increased the marital adjustment of partners compared to the control group (SMD 0.53, 95% CI 0.20 to 0.86; 4 trials, 347 partners, very low certainty). In contrast, non-theory-based couple-based interventions did not significantly influence the marital adjustment of partners compared to the control group (SMD − 0.15, 95% CI -0.48 to 0.18; 3 trials, 162 partners, very low certainty).

#### Marital satisfaction of partners

**Fig. 8:**
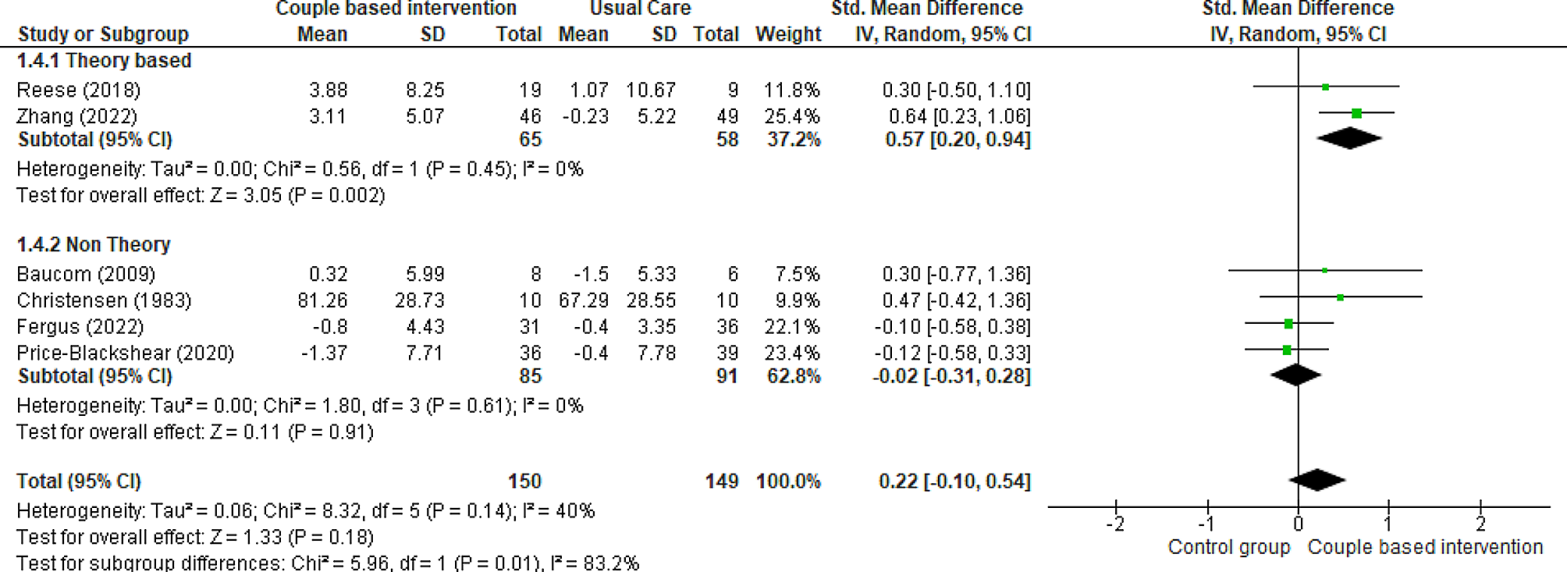
Couple-based intervention group versus control group, Outcome 5: Marital satisfaction of partners

Six RCTs [19, 23, 24, 36, 40, 42] compared the marital satisfaction of intervention groups (receiving couple-based education) with control groups (receiving routine care or general education or a waitlist) in intimate partners of patients. To evaluate marital satisfaction, Fergus et al. used the Kansas Marital Satisfaction Survey [19], Zhang et al. utilized the Olson Marital Quality Questionnaire [23], two studies employed the QMI [24, 36], Reese et al. applied the PROMIS SexFS [40], Christensen et al. used the SSS [42], and Kalaitzi et al. utilized a sexuality and body image tool [43]. The results of most studies showed that couple-based interventions improved the marital satisfaction of partners compared to the control group [23, 36, 40, 42]. However, the results of one study indicated no changes in the marital satisfaction of partners [19], and the opposite effect was observed in another study [24]. All these studies were included in the meta-analysis. Data obtained from six studies performed on 299 partners disclosed that the coupled-based intervention could not affect marital satisfaction compared with the control group, but the evidence is uncertain (Fig. 8) (SMD 0.22, 95% CI -0.10 to 0.54; 6 trials, 299 patients, very low certainty). The result of meta-analysis with excluding studies that the control group received general education showed that there was no change in the significance of the result (SMD 0.32, 95% CI -0.01 to 0.66; 5 trials, 224 patients, very low certainty).

The subgroup analysis results demonstrated that theory-based couple-based interventions increased the marital satisfaction of partners compared to the control group (SMD 0.57, 95% CI 0.20 to 0.94; 2 trials, 123 partners, very low certainty). In contrast, non-theory-based couple-based interventions had no significant effect on the marital satisfaction of partners compared with the control group (SMD − 0.02, 95% CI -0.31 to 0.28; 4 trials, 176 partners, very low certainty).

#### Marital intimacy of partners

**Fig. 9:**
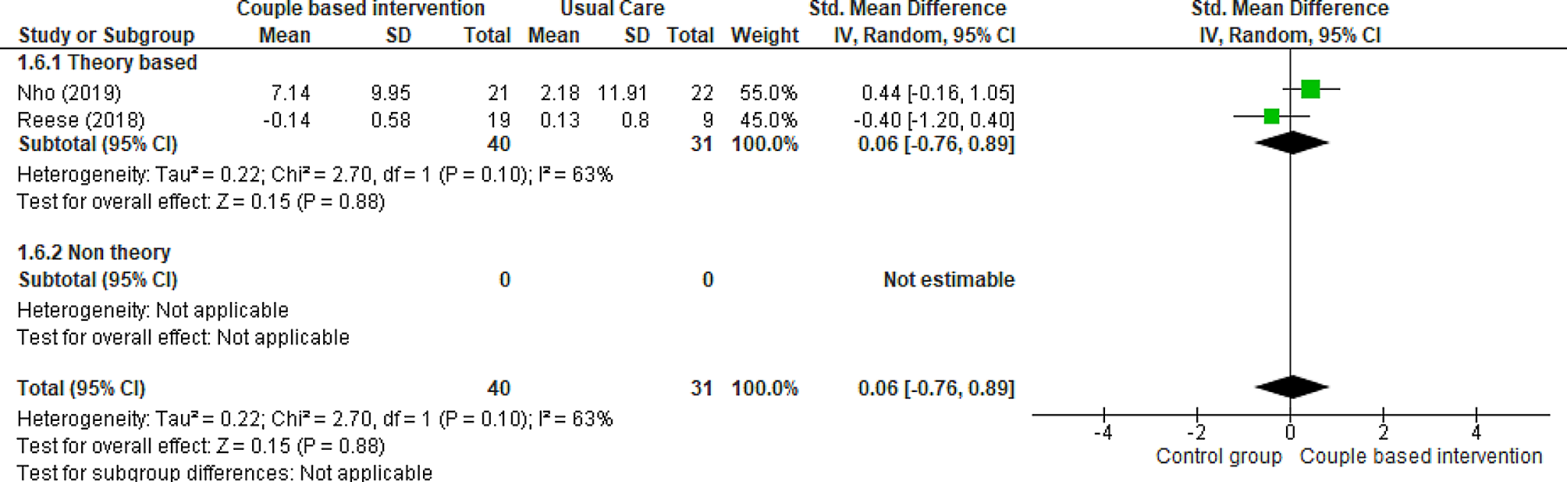
Couple-based intervention group versus control group, Outcome 6: Marital Intimacy of partners

One RCT [40] and three quasi-experimental trials [17, 38, 41] compared marital intimacy in intimate partners of patients in both intervention (receiving couple-based education) and control (receiving routine care) groups. To evaluate marital intimacy, Reese et al. used the PAIR questionnaire [40], Jonsdottir et al. utilized the Ice-Beliefs Questionnaire [17], Nho et al. employed the Marital Intimacy Tool [38], and Hedayati et al. applied the Marital Intimacy Questionnaire Bagarozzi [41]. The results of two studies showed that couple-based interventions led to a significant increase in marital intimacy between couples [17, 41]. In a study by Nho et al., marital intimacy significantly increased between intimate partners [38]. Although Hedayati et al. reported marital intimacy based on couples, they did not report it separately by patients and intimate partners. Additionally, Jonsdottir et al. reported the results of control and intervention groups with each other, thus these two studies were not included in the meta-analysis. A meta-analysis applied to data from two trials performed on 71 patients indicated that couple-based interventions had no effect on the marital intimacy of partners compared to routine care, but the evidence is uncertain (Fig. 9) )SMD 0.06, 95% CI -0.76 to 0.89; 2 trials, 71 patients, very low certainty).

According to the quality or certainty of evidence evaluated using the GRADE approach, the quality of evidence decreased by three degrees and reached a very low certainty in marital adjustment outcomes of patients and partners and patients’ marital satisfaction due to the serious concern about evaluating the risk of bias and inconsistency in the included studies. In the marital intimacy outcome of patients, the quality of evidence was reduced by three degrees and reached very low certainty due to the serious concern about evaluating the risk of bias and imprecision in the included studies. In the marital satisfaction outcome of the partner and marital intimacy of the partner, the quality of evidence was reduced by three degrees and reached very low certainty due to the severe concern about evaluating the risk of bias, inconsistency, and imprecision (Table 5).

**Table 5:** Certainty of the evidence using the GRADE approach by outcomes

No of studies	Design	Risk of bias	Inconsistency	Indirectness	Imprecision	Publication bias	Couple-based intervention	Routine care	Pooled effect Relative (95% CI)	Final judgment
Marital adjustment (Patients)										
7	RCT^*^ Semi-experimental	Serious	Very Serious	No serious	No serious	No serious	268	251	SMD 1.6 Upper (0.6 upper to 2.7 upper)	⊕◯◯◯ Very low
Marital satisfaction (Patients)										
7	RCT^*^	Serious	Very Serious	No serious	No serious	No serious	170	171	SMD 0.6 upper (0.1 upper to 1.1 upper)	⊕◯◯◯ Very low
Marital Intimacy (Patients)										
2	RCT^*^ Semi-experimental	Serious	No serious	No serious	Very serious	No serious	40	31	SMD 0.06 upper (0.5 lower to 0.6 upper)	⊕◯◯◯ Very low
Marital adjustment (Partners)										
7	RCT^*^ Semi-experimental	Serious	Very Serious	No serious	No serious	No serious	262	247	SMD 1.6 upper (0.6 upper to 2.7 upper)	⊕◯◯◯ Very low
Marital satisfaction (Partners)										
6	RCT^*^	Serious	Serious	No serious	Serious	No serious	150	149	SMD 1.1 upper (0.1 Lower to 2.3 upper)	⊕◯◯◯ Very low
Marital Intimacy (Partners)										
2	RCT^*^ Semi-experimental	Serious	Serious	No serious	Very serious	No serious	40	31	SMD 0.2 lower (0.8 Lower to 0.3 upper)	⊕◯◯◯ Very low

**CI**: confidence interval; **RCT**: randomized controlled trial; **SMD**: standardized mean difference

**GRADE Working Group grades of evidence**

**High certainty**: we are very confident that the true effect lies close to that of the estimate of the effect.

**Moderate certainty**: we are moderately confident in the effect estimate; the true effect is likely to be close to the estimate of the effect, but there is a possibility that it is substantially different.

**Low certainty**: our confidence in the effect estimate is limited; the true effect may be substantially different from the estimate of the effect.

**Very low certainty**: we have very little confidence in the effect estimate; the true effect is likely to be substantially different from the estimate of effect.

### Marital relationship of partners

No studies were found regarding the effect of couple-based interventions on marital relationships.

## Discussion

The results of this systematic review of 10 RCTs and four quasi-experimental trials demonstrated that, compared with no intervention, couple-based interventions might increase patients’ marital satisfaction (providing routine care, general education or no intervention); however, the evidence is uncertain. However, there were no significant differences between the groups in outcomes such as the marital satisfaction of partners, marital adjustment, and marital intimacy between patients and partners. On the other hand, the results of the subgroup analysis showed that the marital satisfaction and marital adjustment of patients and partners increased significantly compared to the control group in studies that used couple-based interventions with a theoretical basis or conceptual framework for the intervention. In contrast, no significant difference between the intervention and control groups was observed in the studies that did not follow a specific conceptual framework.

Regarding the outcome of marital satisfaction, Wang et al. performed a systematic review of 12 RCTs to evaluate the effectiveness of couple-based interventions in the health-related quality of life (including marital satisfaction and depression and anxiety) in patients with cancer and their spouses. The results of the study showed that couple-based interventions significantly improved marital satisfaction and reduced depression and anxiety in the patients and their spouses. The result of this study is in line with those of the current study [44]. Li et al. conducted a systematic review of couple-based interventions on couples coping with cancer by including 12 RCTs and five cohort studies. In their study, the patients suffered from any kind of cancer, including prostate, breast, and digestive cancers. The results showed improvements in marital satisfaction and sexual performance in the patients and their partners, which corresponds to the findings of the current study [45]. However, only one of the included studies in these two systematic reviews was specific to the outcome of marital satisfaction in breast cancer patients, and the remaining studies were related to different types of cancer. Considering that breast and genital cancer, which affect femininity, can have a greater impact on marital satisfaction [46, 47], On the other hand, marital satisfaction is an issue related to couples, which highlights the importance of couple interventions in this type of cancer patients and their intimate partners.

Regarding our findings about subgroup analysis and theory-based intervention, the results of a systematic review showed that a web-based training program based on Roy’s theory improved couples’ marital adjustment. This finding shows the importance of using theory in interventions [48]. To fully realize the potential of health services research in enhancing healthcare delivery, it is recommended that institutions and researchers prioritize the integration of theory [49]. Studies indicate that incorporating theory as the foundation for interventions leads to greater changes in health behaviors compared to interventions without a theoretical basis [50]. Couple-based interventions, which are rooted in theory and conceptual frameworks, offer a structured approach to address the unique needs of couples [51]. Li et al.‘s study emphasizes the significance of developing a conceptual framework for couple-based interventions in cancer patients and their intimate partners. This study combines the theories used in the included studies and presents a preliminary Live With Love Conceptual Framework (P-LLCF) theory for cancer couples [52]. In another study by Manne et al., the authors emphasized the importance of using theory in the interventions of couples facing cancer. In this study, resource theories such as cognitive-social processing theory explained how marital relationships can provide support for both patients and partners during challenging life events such as cancer [53]. It seems that by utilizing theory, interventions can target specific aspects of the couple’s relationship, communication patterns, coping strategies, and emotional expression, thereby increasing the likelihood of improving marital outcomes.

In our study, we found a nonsignificant difference in marital intimacy outcome, possibly because of the low number of included studies and patients. This can be described by the very small sample volume of the included studies to determine the effectiveness of the intervention. On the other hand, Hedayati et al.‘s study reported the positive effect of a couple-based intervention on couples’ marital intimacy. However, considering that the results were reported for the couple (not for the patient and partner separately), it was not included in the meta-analysis [41]. Therefore, it seems that more intervention studies are needed in this regard to help the findings of the current study.

Overall, experiencing a cancer diagnosis and undergoing treatment can significantly impact not only the individuals directly affected but also their intimate partners. Cancer can strain even the strongest relationships, leading to increased conflict, decreased intimacy, and reduced satisfaction. Considering the significant impact that a cancer diagnosis and treatment can have on both individuals and their intimate partners, it is crucial to consider couple-based interventions. These findings may indicate that implementing couple-based interventions is more needed in patients with breast and genital cancers than in those with other types of cancer.

### Strengths and limitations

The strengths of the current study are the use of the Cochrane Handbook for the Systematic Review of Trials and the study registration in PROSPERO. The studies were searched in two steps, at the beginning and before the end of the study, and no limitations were applied to the publication dates of the studies. Additionally, almost all the studies mentioned the outcomes of partners, except for one study that focused only on the outcomes of patients. Regarding the limitations of this study, the included studies were limited to the English and Persian languages. In addition, only three studies were performed on patients with genital cancer and their intimate partners, and the rest were related to breast cancer. Therefore, additional studies should be conducted in this context to help confirm the findings of the present study. Additionally, the conclusion was limited due to the very low-certainty evidence.

## Conclusion

According to the meta-analysis results, couple-based interventions according to the theoretical context are effective at improving the marital outcomes of patients with breast and genital cancers and their partners, but the evidence is uncertain. The results of this systematic review indicate that few studies are available about the effect of couple-based interventions on some outcomes, such as marital intimacy. Therefore, high-quality RCTs and sufficient sample volumes should be carried out based on the CONSORT statement and a useful theoretical context to clarify the impact of couple-based education on these outcomes. Additionally, couple-based interventions for male cancer patients and their intimate partners are recommended for further studies.

### Electronic supplementary material

Below is the link to the electronic supplementary material.

Supplementary Material 1

## Data Availability

All the data are included in the tables.
