# Supplementary material for: The effectiveness of couple-based interventions on the marital outcomes of women with genital and breast cancer and their partners: a systematic review and meta-analysis
Source: BMC Cancer. 2024 Mar 27;24:391. doi: 10.1186/s12885-024-12088-x (PMC10976738; doi:10.1186/s12885-024-12088-x)
Supplement: Supplementary file 1 — Supplementary Material 1 [file 12885_2024_12088_MOESM1_ESM.docx]

**Search Strategy:**

Systematic searches of **Scopus** database were performed beginning on 30^th^ April 2023 with related keywords to obtain published studies in English using below search strategy:

( ( ( TITLE-ABS-KEY ( "gynecologic* cancer" ) OR TITLE-ABS-KEY ( "gynecologic* neoplasm" ) OR TITLE-ABS-KEY ( "gynecologic* tumor" ) OR TITLE-ABS-KEY ( "gynecologic* carcinoma" ) OR TITLE-ABS-KEY ( "genital cancer" ) OR TITLE-ABS-KEY ( "genital neoplasm" ) OR TITLE-ABS-KEY ( "genital tumor" ) OR TITLE-ABS-KEY ( "genital carcinoma" ) OR TITLE-ABS-KEY ( "ovarian cancer" ) OR TITLE-ABS-KEY ( "ovarian neoplasm" ) OR TITLE-ABS-KEY ( "ovarian tumor" ) OR TITLE-ABS-KEY ( "ovarian carcinoma" ) OR TITLE-ABS-KEY ( "uterine cancer" ) OR TITLE-ABS-KEY ( "uterine neoplasm" ) OR TITLE-ABS-KEY ( "uterine tumor" ) OR TITLE-ABS-KEY ( "uterine carcinoma" ) OR TITLE-ABS-KEY ( "cervical cancer" ) OR TITLE-ABS-KEY ( "cervical neoplasm" ) OR TITLE-ABS-KEY ( "cervical tumor" ) OR TITLE-ABS-KEY ( "cervical carcinoma" ) OR TITLE-ABS-KEY ( "endometrial cancer" ) OR TITLE-ABS-KEY ( "endometrial neoplasm" ) OR TITLE-ABS-KEY ( "endometrial tumor" ) OR TITLE-ABS-KEY ( "endometrial carcinoma" ) OR TITLE-ABS-KEY ( "Vaginal cancer" ) OR TITLE-ABS-KEY ( "Vaginal neoplasm" ) OR TITLE-ABS-KEY ( "Vaginal tumor" ) OR TITLE-ABS-KEY ( "Vaginal carcinoma" ) OR TITLE-ABS-KEY ( "Vulvar cancer" ) OR TITLE-ABS-KEY ( "Vulvar neoplasm" ) OR TITLE-ABS-KEY ( "Vulvar tumor" ) OR TITLE-ABS-KEY ( "Vulvar carcinoma" ) ) ) OR ( ( TITLE-ABS-KEY ( "breast cancer" ) OR TITLE-ABS-KEY ( "breast neoplasm" ) OR TITLE-ABS-KEY ( "breast tumor" ) OR TITLE-ABS-KEY ( "breast carcinoma" ) OR TITLE-ABS-KEY ( mastectomy ) ) ) ) AND ( ( ( TITLE-ABS-KEY ( "marital satisfaction" ) OR TITLE-ABS-KEY ( "relationship satisfaction" ) OR TITLE-ABS-KEY ( "couple satisfaction" ) OR TITLE-ABS-KEY ( "Spousal satisfaction" ) OR TITLE-ABS-KEY ( "sexual satisfaction" ) ) ) OR ( ( TITLE-ABS-KEY ( "marital adjustment" ) OR TITLE-ABS-KEY ( "couple adjustment" ) OR TITLE-ABS-KEY ( "dyadic adjustment" ) OR TITLE-ABS-KEY ( "sexual adjustment" ) OR TITLE-ABS-KEY ( "marital adaptation" ) OR TITLE-ABS-KEY ( "couple adaptation" ) OR TITLE-ABS-KEY ( "dyadic adaptation" ) OR TITLE-ABS-KEY ( "sexual adaptation" ) ) ) OR ( ( TITLE-ABS-KEY ( "marital relationship" ) OR TITLE-ABS-KEY ( "couple relationship" ) OR TITLE-ABS-KEY ( "dyadic relationship" ) OR TITLE-ABS-KEY ( "sexual relationship" ) OR TITLE-ABS-KEY ( "couple interaction" ) OR TITLE-ABS-KEY ( "marital interaction" ) ) ) OR ( ( TITLE-ABS-KEY ( "Marital Intimacy" ) OR TITLE-ABS-KEY ( "sexual intimacy" ) ) ) ) AND ( ( TITLE-ABS-KEY ( partner ) OR TITLE-ABS-KEY ( spouse ) OR TITLE-ABS-KEY ( couple ) OR TITLE-ABS-KEY ( husband ) OR TITLE-ABS-KEY ( dyad ) OR TITLE-ABS-KEY ( partnership ) ) ) AND ( ( TITLE-ABS-KEY ( "Couple therapy" ) OR TITLE-ABS-KEY ( "couple-based intervention" ) OR TITLE-ABS-KEY ( "couple-oriented intervention" ) OR TITLE-ABS-KEY ( "couple-focused intervention" ) OR TITLE-ABS-KEY ( "dyadic intervention" ) OR TITLE-ABS-KEY ( "supportive care intervention" ) OR TITLE-ABS-KEY ( program ) OR TITLE-ABS-KEY ( training ) OR TITLE-ABS-KEY ( "problem solving" ) OR TITLE-ABS-KEY ( counsel* ) OR TITLE-ABS-KEY ( education ) OR TITLE-ABS-KEY ( intervention ) OR TITLE-ABS-KEY ( support ) ) ) AND ( ( TITLE-ABS-KEY ( "RCT" ) OR TITLE-ABS-KEY ( "Randomized controlled trial" ) OR TITLE-ABS-KEY ( "Randomised controlled trial" ) OR TITLE-ABS-KEY ( "clinical trial" ) OR TITLE-ABS-KEY ( experimental ) OR TITLE-ABS-KEY ( randomization ) ) )
